# Poor sleep quality and associated factors among HIV-positive and negative postpartum women in Addis Ababa, Ethiopia: a comparative cross-sectional study

**DOI:** 10.3389/fpsyt.2024.1362384

**Published:** 2025-01-23

**Authors:** Milen Mihertabe, Alehegn Bishaw Geremew, Elsa Awoke Fentie, Gidey Rtbey

**Affiliations:** ^1^ Department of Psychiatry, College of Medicine and Health Sciences, University of Gondar, Gondar, Ethiopia; ^2^ Department of Reproductive Health, Institute of Public Health, College of Medicine and Health Sciences, University of Gondar, Gondar, Ethiopia; ^3^ Flinders Health and Medical Research Institute, College of Medicine and Public Health, Flinders University, Adelaide, SA, Australia

**Keywords:** poor sleep quality, postpartum women, human immunodeficiency virus, Pittsburgh Sleep Quality Index, Ethiopia

## Abstract

**Background:**

Poor sleep quality is common during the postpartum period due to nighttime feeding and frequent nocturnal awakenings of infants. In addition, the condition may worsen among postpartum women living with human immunodeficiency virus (HIV), affecting their capacity to care for their child. Therefore, this study will provide baseline information by assessing the burden of poor sleep quality and associated factors among HIV-positive and negative postpartum women in Addis Ababa, Ethiopia.

**Objective:**

The aim of this study was to assess poor sleep quality and its associated factors among HIV-positive and negative postpartum women in Addis Ababa, Ethiopia in 2023.

**Methods:**

A comparative cross-sectional study was conducted from 20 March to 20 May 2023, and a systematic random sampling technique was employed to obtain a total of 535 study subjects. An interviewer-administered questionnaire and chart review were used to collect the data. The Pittsburgh Sleep Quality Index was used to assess the outcome variable with a cutoff point ≥5. Binary logistic regression was employed to check the association of each independent variable with the outcome variable. Variables having a *p*-value of less than 0.05 in multivariable analysis were considered statistically significant and an adjusted odds ratio with a 95% confidence interval (CI) was computed.

**Results:**

In this study, the overall prevalence of poor sleep quality among postpartum women was 56.3% [95% CI (51.9, 60.6)]. Poor sleep quality prevalence among HIV-positive and -negative women was 80% and 50.5%, respectively. Being HIV-positive [AOR = 2.38, 95% CI (1.31, 4.32)], being divorced [AOR = 4.5, 95% CI (1.55, 13)], and having a family history of mental illness [AOR = 1.89, 95% CI (1.16, 3.1)], depression [AOR = 1.93 (1.11, 3.3)], and anxiety [AOR = 2.76, 95% CI (1.8, 4.2)] among postpartum women and poor social support [AOR = 6, 95% CI (1.63, 22.4)] among HIV-positive women were significantly associated with poor sleep quality.

**Conclusion and recommendation:**

This study revealed that the prevalence of poor sleep quality was higher among HIV-positive postpartum women compared to HIV-negative women. It would be better if professionals routinely assess postpartum women for sleep quality and focus on HIV-positive women.

## Introduction

Sleep is a natural process that the brain requires for it to continue to properly function and maintain good health. Sleep takes up one-third of human life and is a significant contributor to energy restoration for proper functioning (1, 2). Sleep quality is defined as a person’s satisfaction with different sleep experiences including sleep efficiency, latency, duration, and wake after sleep onset (2, 3).

A global study revealed that sleep disturbance in the general population was 23.5% (4), and a study conducted in Germany indicated that poor sleep quality prevalence was 36% with a higher prevalence in women (5). The physiological, psychological, and social changes during the postpartum period impact sleep quality (6). According to the American College of Obstetricians and Gynecologists (ACOG), the postpartum period encompasses not only physical recovery but also the establishment of breastfeeding, mental health considerations (such as postpartum depression), and other aspects of maternal–infant bonding that can affect sleep quality (7, 8). Sleep-related problems, such as poor sleep quality, often occur in perinatal women, with 14% to 76% experiencing clinically significant insomnia symptoms (9, 10), and this figure increases up to 87.5% in postpartum women (6). A systematic review conducted in China among pregnant and postpartum women revealed that 44.5% of pregnant and 67.2% of postpartum women experience poor sleep quality (11).

Acquired immune deficiency syndrome (AIDS) is one of the most serious health issues globally, particularly in developing nations (12). More than 36.9 million individuals worldwide are afflicted with HIV/AIDS, and sub-Saharan Africa is the most highly affected region, with an estimated 1 in every 25 people living with the virus (13, 14).

Sleep-related problems are common manifestations of people with human immunodeficiency virus (HIV). A global meta-analysis reported that the prevalence of sleep disturbances among people living with HIV was 58% (15), and a study in sub-Saharan African countries revealed that the pooled prevalence of poor sleep quality among people living with HIV was 49.32% (16). Studies in Ethiopia show that the prevalence of poor sleep quality in postpartum women ranges between 21.8% (17) and 24% (18). Though there was no study conducted on sleep quality among HIV-positive postpartum women in Ethiopia, a study in HIV-positive antenatal women indicates that poor sleep quality prevalence was 39.4% (19). Poor sleep quality in people with HIV could be due to a direct effect of the virus and opportunistic infection of the central nervous system (CNS) (20).

Different studies revealed that prolonged sleep deprivation could lead to severe physical illness and the development of mental health problems like substance use disorders and cognitive impairments (1). Poor sleep quality in the postpartum period is linked to serious health problems that strike mothers at a crucial time in their lives and can have a variety of detrimental effects on their partners besides causing emotional, behavioral, and sleep issues in newborns (17). Moreover, women who experience poor sleep quality during the postpartum period are more susceptible to anxiety and depression later in life (21). A study on depression conducted in postpartum women revealed that poor sleep quality in the early postpartum period independently predicts the development of later postpartum depression (22).

As several studies have shown, physical discomfort, changes in household responsibilities, financial strains, and a lack of time due to increased caregiving responsibilities are among the factors that contribute to poor sleep quality during the postpartum period (23). Women experience changes in their sleeping patterns and increased levels of tiredness after childbirth (24). For instance, postnatal women typically get less sleep and have a worse quality of sleep in the early postpartum period compared to when they were pregnant or in earlier reproductive age periods (prior to their pregnancy) (25). HIV’s ability to affect the CNS, opportunistic infections, the pharmacological effects of antiretroviral drugs like efavirenz (EFV), and mental health problems in people living with HIV can negatively affect their sleep quality (20, 26, 27). Furthermore, having depressive and anxiety symptoms, poor social support, a CD4 count of less than 200 cells/mm**^3^**, and viral loads of higher than 1,000 copies are factors associated with poor sleep quality in people living with HIV (19, 26, 28). The sleep problem in postpartum women is worse for those who underwent cesarean section as a result of post-cesarean section pains (29).

Despite having such a huge negative impact on women’s life multidimensionally, little attention is given to sleep problems. Most previously conducted studies in Ethiopia were focused on pregnancy only, in which postpartum was not the focal point. Therefore, this study, besides assessing the sleep quality and associated factors among HIV-positive and -negative postpartum women, compared the problem between the two segments of the population. The findings of the current study can be helpful for professions working in postnatal care clinics, future researchers, and responsible officials to plan strategies in tackling the problem.

## Methods and materials

### Study design, setting, and period

An institutional-based comparative cross-sectional study was conducted at Comprehensive Specialized Hospitals in Addis Ababa. Addis Ababa is the capital city of Ethiopia and the seat of the African Union. It has seven Comprehensive Specialized Hospitals serving approximately 5.4 million inhabitants. Thus, this study was carried out among HIV-positive and -negative mothers who were attending postnatal care at those Comprehensive Specialized Hospitals from 20 March to 20 May 2023.

### Study participants

All HIV-positive and -negative postpartum women who were attending postnatal care at the Comprehensive Specialized Hospitals in Addis Ababa during the data collection period and postpartum women aged 18 years and above were included in this study. However, HIV-positive and -negative postpartum women who had a serious medical illness (post-operation sepsis or severely ill HIV-positive women) and were unable to communicate were excluded from this study.

### Sample size and sampling procedure

Sample size calculation was done for two objectives; the first objective was determined by using a double population proportion formula by taking an assumption *Za*/2 = 1.96 (type I error), β (type II error) = 0.84, *r* = ratio of sample size *n*_1_ (sample size of HIV-positive postpartum women) to *n*_2_ (sample size of HIV-negative postpartum women), which is taken as 1 to 4, *P*_1_ = proportion of poor sleep quality among HIV-positive postpartum mothers, and *P*_2_ = proportion of poor sleep quality among HIV-negative postpartum mothers. Since there was no study conducted among HIV-positive postpartum women, *P*_1_ was taken as 39.4% from a study conducted among HIV-positive pregnant women in northwest Ethiopia (19), whereas in the case of this study, the value of *P* **_2_** was 24% from the study conducted in Gondar city (18).


$$\begin{array}{c} n=\frac{\left(Z@/2+\text{β}\right)2\left(\text{P}1\text{q}1+\;\text{p }2\text{q }2\right)}{\left(\text{P}1–\text{P}2\right)2},n\\ =\frac{\left(1.96+0.84\right)2\left(0.394*0.606+0.76*0.24\right)}{\left(0.394-0.24\right)2}\;=193 \end{array}$$

The sample size calculation for the second objective was performed using the Epi info software version-7 employing different variables that have contributed to poor sleep quality in previous studies. During the sample size calculation using Epi info, the following assumptions were considered: 95% confidence interval, the ratio of unexposed and exposed (*r* =1), and 80% power. The sample size for the second objective was found to be higher than the first objective. Finally, the second objective’s sample size for the current study was 535 after adding 10% of the non-response rate.

### Sampling technique and procedure

A total of 535 postpartum women were recruited. Initially, the sample size was proportionally allocated among the seven Comprehensive Specialized Hospitals based on the data obtained from each hospital’s monthly follow-up of postpartum women before the actual data collection period. In addition to this, proportional allocation of sample size among HIV-positive and -negative postpartum women at each hospital was performed separately. Then, a systematic random sampling technique was employed to draw study subjects from each hospital by calculating the value of “K” for both study populations (HIV-positive and -negative women). The first participant in each hospital was chosen using a lottery method and then continued to recruit participants at a given “K” value. Finally, the proportion of HIV-positive to HIV-negative postpartum women was taken at approximately 1:4.

## Study variables

### Dependent variable

Poor sleep quality.

### Independent variables

Sociodemographic data: Age, educational status, residence, religion, occupation, and marital status.

Gynecological and obstetric-related factor: Pregnancy type and mode of delivery.

Behavioral, psychosocial factors, and intimate partner violence: Substance use like caffeine, khat, alcohol, tobacco smoking, and others, anxiety, depression, social support, intimate partner violence, and family history of mental illness.

HIV-related variables: prevention of mother-to-child transmission (PMTCT) follow-up, clinical stage, viral load, and cluster of differentiation 4 (CD4) count. Clinical factors: Known chronic medical illness other than HIV/AIDS.

### Data collection tools and procedure

Data were collected using a pre-tested interviewer-administered questionnaire and chart review. The questionnaire was developed in English and translated into Amharic then translated back to English by different experts to check its consistency. The data were collected from seven midwives, facilitated by two psychiatry professionals and the principal investigator. The principal investigator supervised and provided all necessary items for the data collection on each data collection day, checking the filled out questionnaire for completeness, and solving problems forwarded during the data collection period.

The questionnaire had six sections. The first section consisted of sociodemographic data, developed after reviewing different literature, providing baseline information on study participants.

Section 2 included the Pittsburgh Sleep Quality Index (PSQI) assessment tool of the outcome variable. It is a 19-item self-report sleep quality assessment tool within the previous month used to measure maternal sleep quality during the postpartum period. PSQI consists of seven component scores (ranging from 0 to 3), measuring subjective sleep quality, sleep latency, sleep duration, sleep efficiency, sleep disturbances, use of sleeping medication, and daytime dysfunction. A global PSQI score, which ranges from 0 to 21, is calculated by adding the scores of the seven components’ higher scores indicating higher overall sleep disturbances. The instrument was validated in Ethiopia among community-dwelling adults with sensitivity and specificity values of 82% and 56.2%, respectively, at a cutoff point of 5.5 for screening insomnia (30). In the current study, postpartum women who scored 5 and above in PSQI were considered to have poor sleep quality, referring to a previous study conducted in Ethiopia (18). A global PSQI score of 5 and above indicates poor sleep quality (31) and the reliability coefficient of PSQI in the current study was found to be 0.88.

Section 3 included the EPDS postpartum depression assessment tool. A standardized 10-item questionnaire with scores ranging from 0 to 3 is used to assess depression in the pre- and postpartum periods. The tool was validated among postpartum women in public health centers in Addis Ababa, Ethiopia. At the cutoff point of 7/8, the instrument demonstrated a sensitivity of 84.6% and a specificity of 77% (32). Postpartum women who scored 8 and above for EPDS in this study were considered to have depression (33). The reliability coefficient of EPDS in the current study was high (α = 0.83).

Section 4 comprised the Generalized Anxiety Disorder 7-item (GAD-7) scale. It is a self-reporting questionnaire consisting of seven items measuring the frequency of anxiety-related symptoms experienced over the past 2 weeks. Each item is rated on a scale from 0 to 3, with the following options for responses. The total score ranges from 0 to 21, with higher scores indicating severity of anxiety symptoms (34). However, the GAD-7 scale is not validated in the Ethiopian context, and the Spanish-language version was tested as a reliable (α = 0.89) screening tool for postnatal anxiety with a cutoff point of 5 and above (35). Postpartum women who scored 5 and above in GAD-7 were screened positive for anxiety in this study and the reliability coefficient of the tool was acceptable (α = 0.798).

Section 5 included abuse assessment screening for intimate partner violence and Oslo-3 social support. A current spouse, cohabited, current boyfriend, former partner, or spouse is considered to be an intimate partner. A woman was considered to have experienced intimate partner violence if she reported experiencing anyone of the ranges of sexual, psychological, and physical or any combination of the three coercive acts regardless of the legal status of the relationship with her current/former intimate partner (36). The Oslo Social Support Scale (OSS-3) scores ranged from 3 to 14 with a score of 3–8 indicating poor social support, 9–11 denoting moderate social support, and 12–14 indicating strong social support (37).

Section 6 consisted of behavioral and clinic-related factors with simply structured questions, and current substance use was assessed using the Alcohol, Smoking, and Substance Involvement Screening Test (ASSIST) tool. Current substance use was assessed by a simple question, “Have you used any substance in the last 3 months for non-medical use?” (38). The last section included HIV-related factors and was exclusively for HIV-positive postpartum women.

### Data quality control

Pre-data collection training was provided for data collectors, and appropriately designed data collection instruments were constructed to ensure data quality. Before any actual data were gathered, 5% of the sample size of 27 women were pre-tested using the questionnaire at Zewditu Referral Hospital. The principal investigator verified the completeness of all the data gathered on each data collection day.

### Data processing and analysis

Data entry was carried out using Epi Data version 4.6.02 and then exported to STATA version 14 for analysis. Using STATA, coding, cleaning, and analysis were completed. The adjusted odds ratios with 95% confidence intervals were computed using binary logistic regression analysis. Pearson’s chi-square test was employed to evaluate the association between HIV status and sleep quality. Initially, each independent variable was checked for its association with the outcome variable, and variables with a p-value of < 0.25 in a bivariable analysis were candidates to be included in multivariable analysis. Before performing multivariable logistic regression, each variable’s variable inflation factor (VIF) was calculated to assess the multicollinearity, and the values were less than five, indicating no significant multicollinearity. Potential confounding variables like age were checked by including them in the binary logistic regression model to identify the effect of these variables on the final association regardless of their significance in bivariable analysis. Variables with a *p*-value of<0.05 in multivariable analysis were declared statistically significant with the outcome variable. An adjusted odds ratio with a 95% confidence interval was computed to see the presence, strength, and direction of association between dependent and independent variables. Firstly, the regression analysis was conducted to identify factors associated with poor sleep quality among overall postpartum women, considering HIV status as an independent variable. Second, analysis was performed for HIV-positive and HIV-negative postpartum women separately, intending to reveal factors in the two groups of population. The goodness-of-fit model was checked using Hosmer and Lemeshow’s goodness of fit and it was fit.

## Results

### Sociodemographic characteristics of study participants

A total of 510 participants fully responded to the questionnaires, yielding a response rate of 95.3%. The mean age of respondents was 30.57 years (SD ± 0.26). Most of the women were HIV-negative 410 (80.39%), 389 (76.27%) of whom were married and 66 (12.94%) were single. Approximately 193 (37.84%) participants attended primary school, followed by 160 (31.37%) who attended secondary school. Approximately 254 (49.8%) were Orthodox, 103 (20.2%) were Muslims, and 463 (90.78%) had an urban residence (**Table 1**).

**Table 1:** Sociodemographic characteristics of postpartum women attending PNC follow-up at Comprehensive Specialized Hospitals in Addis Ababa, 2023 (*n* = 510).

Variables	Categories	HIV status	
		Negative (*n* = 410)	Positive (*n* = 100)
		Frequency (%)	Frequency (%)
Age	18–24 25–34 35–42	96 (23.4) 230 (56.1) 84 (20.5)	5 (5) 48 (48) 47 (47)
Residence	Urban Rural	374 (91.2) 36 (8.8)	89 (89) 11 (11)
Marital status	Married Single Divorced Widowed	329 (80.3) 45 (10.97) 20 (4.9) 16 (3.9)	60 (60) 21 (21) 11 (11) 8 (8)
Educational status	Cannot/can read write Primary school Secondary school Diploma and above	57 (13.9) 158 (38.5) 128 (31.22) 67 (16.34)	20 (20) 35 (35) 32 (32) 13 (13)
Religion	Orthodox Muslim Catholic Protestant Others*	194 (47.32) 84 (20.49) 54 (13.17) 53 (12.93) 25 (6.1)	60 (60) 19 (19) 5 (5) 13 (13) 3 (3)
Occupational status	Self-employed Government employed Unemployed Housewife Others**/student	79 (19.27) 90 (21.95) 122 (29.75) 93 (22.68) 26 (6.34)	30 (30) 17 (17) 34 (34) 17 (17) 2 (2)

*Others: Pagan and Behay.

**Others: commercial sex workers and farmer.

Secondary school: Junior and senior high school; Diploma and above: College diploma degree and above.

### Psychosocial, substance, and clinical-related characteristics of participants

Among the participants, 240 (47.06%) reported moderate social support, 107 (20.9%) screened positive for depressive symptoms, and 285 (55.88%) experienced anxiety symptoms. Approximately 121 (23.7%) and 140 (27.45%) participants reported a chronic medical illness and a family history of mental illness, respectively (**Table 2**).

**Table 2:** Psychosocial, substance, and clinical-related characteristics of study participants (*n* = 510).

Variables	Categories	HIV status	
		Negative (*n* = 410)	Positive (*n* = 100)
		Frequency (%)	Frequency (%)
Social support	Poor Moderate Strong	153 (37.3) 208 (50.7) 49 (12)	61 (61) 32 (32) 7 (7)
Depression	Yes No	54 (13.2) 356 (86.8)	53 (53) 47 (47)
Anxiety	Yes No	213 (52) 197 (48)	72 (72) 28928)
Current substance	Yes No	28 (6.8) 382 (93.2)	10 (10) 90 (90)
Chronic medical illness	Yes No	103 (25.1) 307 (74.9)	18 (18) 82 (82)
Family history of mental illness	Yes No	117 (28.5) 293 (71.5)	23 (23) 77 (77)
Wanted pregnancy	Yes No	256 (62.4) 154 (37.6)	54 (54) 46 (46)
Mode of delivery	SVD CS	263 (64.1) 147 (35.9)	75 (75) 25 (25)

NB, spontaneous vaginal delivery; CS, cesarean section.

### Intimate partner violence and HIV-related variables

Among the participants, 138 (27.1%) had ever been emotionally or physically abused, and 86 (86%) HIV-positive women reported PMTCT follow-up. The majority (*n* = 68) of HIV-positive mothers (68%) were in clinical stage I (**Table 3**).

**Table 3:** Intimate partner violence and HIV-related factors among postpartum women attending PNC follow-up at Comprehensive Specialized Hospitals in Addis Ababa, 2023 (*n* = 510).

Variables	Categories	HIV status	
		Negative (*n* = 410)	Positive (*n* = 100)
		Frequency (%)	Frequency (%)
Emotionally/physically abused by a partner	Yes No	108 (26.3) 302 (73.7)	30 (30) 70 (70)
Been hit, slapped, or kicked within the last year	Yes No	32 (7.8) 378 (92.2)	11 (11) 89 (89)
Been hit, slapped, or kicked since pregnancy	Yes No	20 (4.9) 390 (95.1)	3 (3) 97 (97)
Being forced to have sexual activities within the last year	Yes No	8 (2) 402 (98)	4 (4) 96 (96)
Being afraid of a partner or anyone listed above	Yes No	11 (2.7) 399 (97.3)	6 (6) 94 (94)
Having had PMTCT follow-up	Yes No		86 (86) 14 (14)
Viral load	<1,000 ≥1,000		59 (59) 41 (41)
CD4 count	<200 ≥200		20 (20) 80 (80)
Clinical stage	Stage I Stage II Stage III		68 (68) 25 (25) 7 (7)

### Prevalence of poor sleep quality among postpartum women

**Figure 1:**
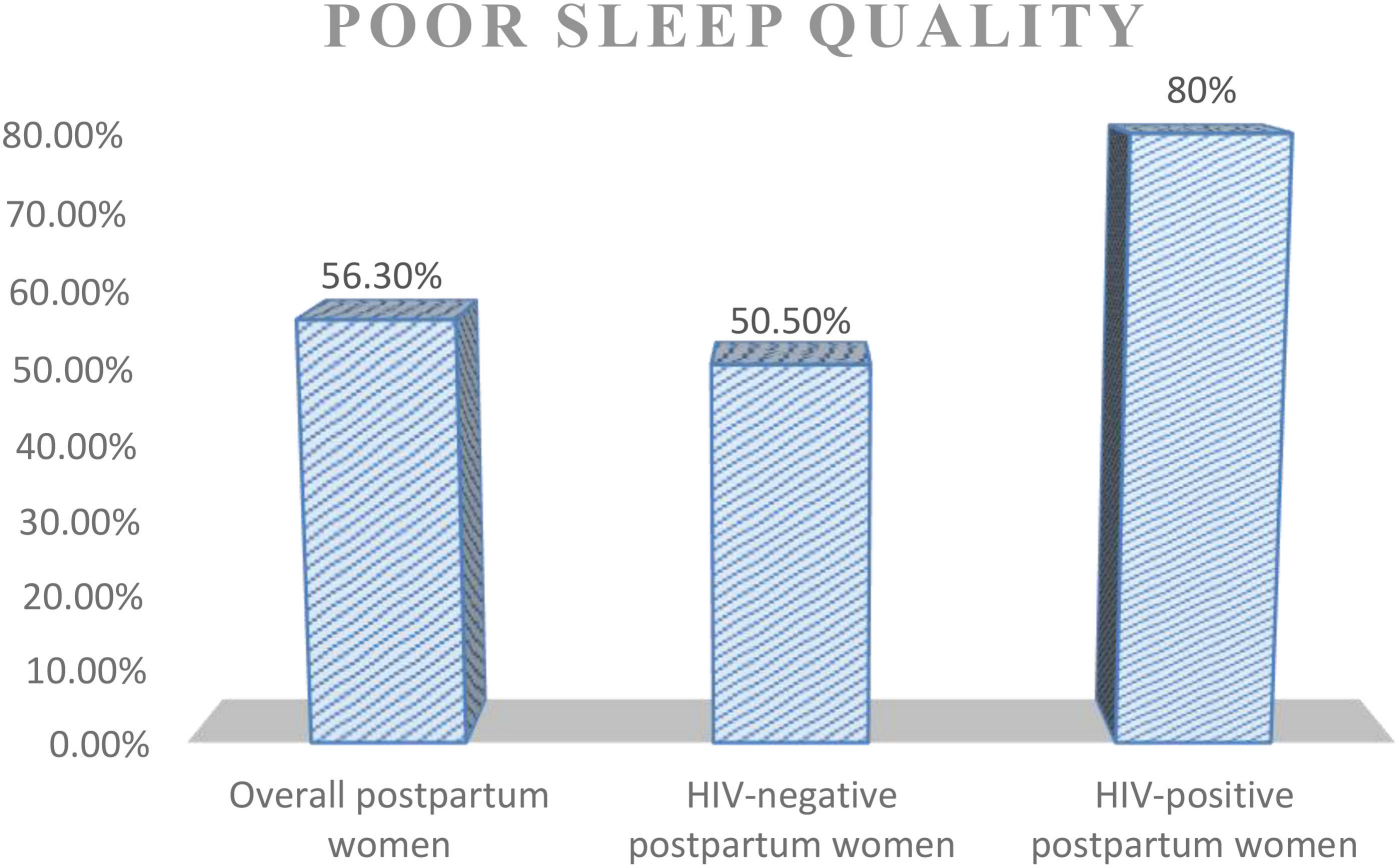
Prevalence of poor sleep quality among women attending PNC at Comprehensive Specialized Hospitals in Addis Ababa, 2023.

The overall prevalence of poor sleep quality among postpartum women was 56.3% [95% CI (51.9, 60.6)], whereas poor sleep quality among HIV-positive and -negative postpartum women was found to be 80% and 50.5%, respectively (**Figure 1**). Approximately 18 (3.5%) postpartum women had very bad subjective sleep quality; as regards sleep duration, 409 (80.2%) reported more than 7 h of actual sleep per night, 169 (33%) participants reported more than 85% sleep efficiency, and 388 (76.1%) reported not using any sleep medications within the last month (**Table 4**).

**Table 4:** PSQI component scores among postpartum women attending PNC follow-up at Comprehensive Specialized Hospitals in Addis Ababa, 2023 (*n* = 510).

PSQI component	Categories	HIV status	
		Negative (*n* = 410) Frequency (%)	Positive (*n* = 100) Frequency (%)
Subjective sleep quality	Very good Fairly good Fairly bad Very bad	79 (19.3) 265 (64.6) 61 (14.9) 5 (1.22)	7 (7) 39 (39) 41 (41) 13 (13)
Sleep latency	0 1–2 3–4 5–6	127 (31) 132 (32.2) 83 (20.2) 68 (16.6)	13 (13) 15 (15) 17 (17) 55 (55)
Sleep duration	>7 h 6–7 h 5–5.59 h<5 h	360 (87.8) 41 (10) 7 (1.7) 2 (0.5)	49 (49) 23 (23) 20 (20) 8 (8)
Habitual sleep efficiency	>85% 75%–84% 65%–74%<65%	149 (36.3) 129 (31.5) 79 (19.3) 13 (12.9)	20 (20) 22 (22) 35 (35) 23 (23)
Sleep disturbance	0 1–9 10–18 19–27	29 (7.1) 339 (82.7) 41 (10) 1 (0.24)	2 (2) 55 (55) 35 (35) 8 (8)
Use of drugs	Not in the past month Less than once a week Once or twice a week Three or more times a week	326 (79.5) 56 (13.7) 27 (6.6) 1 (0.24)	62 (62) 14 (14) 19 (19) 5 (5)
Daytime dysfunction	0 1–2 3–4 5–6	282 (68.8) 94 (22.9) 29 (7.1) 5 (1.22)	29 (29) 18 (18) 25 (25) 28 (28)
**Poor sleep quality**	Yes No	207 (50.5) 203 (49.5)	80 (80) 20 (20)

Sleep latency: Sum of Q**_2_** and Q_5a_ subscores from PSQI and interpreted as 0 = 0, 1–2 = 1, 3–4 = 2, and 5–6 = 3.

Sleep efficiency: (# hours slept/# hours in bed) × 100%.

Sleep disturbance: Sum of questions 5b to 5j from PSQI and scored as follows: not during the past month = 0, less than once a week = 1, once or twice a week = 2, three or more times a week = 3.

Daytime dysfunction: Sum of Q_7_ and Q_8_ subscores from PSQI and interpreted as 0 = 0, 1–2 = 1, 3–4 = 2, and 5–6 = 3.

PSQI, Pittsburgh Sleep Quality Index.

### Chi-square test of sleep quality by HIV status of postpartum women

The chi-square statistic was used to evaluate the association between sleep quality and HIV status at a *p*-value of 0.05. The test indicated that in the current study, there was a significant association between sleep quality and the HIV status of postpartum women (**Table 5**).

**Table 5:** Chi-square test of sleep quality and HIV status (*n* = 510).

HIV status	Sleep quality	
	Good	Poor
Negative	203	207
Positive	20	80

Chi-square statistic (χ²) = 28.4558, *p*-value (Pr) = 0.000.

### Factors associated with poor sleep quality

Overall among postpartum women (HIV-positive and negative women), in the multivariable analysis, being HIV positive, being divorced, and having a family history of mental illness, depression, and anxiety symptoms were significantly associated with poor sleep quality at a *p*-value of<0.05. The odds of reporting poor sleep quality among HIV-positive women was 2.38 times higher compared to those without the virus [AOR = 2.38, 95% CI (1.31, 4.32)], and reporting poor sleep quality among divorced postpartum women was 4.5 times higher compared to married women [AOR = 4.5, 95% CI (1.55, 13)]. The likelihood of experiencing poor sleep quality among postpartum women who had depressive symptoms was approximately two times higher compared to their counterparts [AOR =1.93, 95% CI (1.11, 3.3)]. Postpartum women who had anxiety symptoms were 2.76 times at risk of reporting poor sleep quality than those who did not have anxiety symptoms [AOR = 2.76, 95% CI (1.8, 4.2)]. Having a family history of mental illness was another factor found to be associated with poor sleep quality among postpartum women [AOR = 1.89, 95% CI (1.16, 3.09)] (**Table 6**).

**Table 6:** Bivariable and multivariable analysis of factors associated with poor sleep quality among the overall postpartum women attending PNC at Comprehensive Specialized Hospitals in Addis Ababa, 2023 (*n* = 510).

Variable	Categories	Sleep quality		95% CI	
		Poor	Good	COR	AOR
HIV status	Negative Positive	207 80	203 20	1.00 3.92 (2.31,6.64)	1.00 2.38 (1.31,4.32) **
Marital status	Single Married widowed Divorced	43 202 16 23	23 117 8 5	1.73 (1,2.98) 1.00 1.85 (0.77,4.42) 4.8 (1.8,12.79)	1.12 (0.61,2.05) 1.00 1.34 (0.5,3.56) 4.5 (1.55,13) **
Educational status	Can/cannot read and write Primary school Secondary school Diploma and above	48 108 91 40	29 85 69 40	1.65 (0.87,3.12) 1.28 (0.75,2.14) 1.31 (0.76,2.26) 1.00	1.02 (0.48,2.15) 1.09 (0.58,2.00) 1.35 (0.71,2.5) 1.00
Residence	Urban Rural	267 20	196 27	1.83 (1.00,3.37) 1.00	1.79 (0.89,3.6) 1.00
Wanted pregnancy	Yes No	160 127	150 73	1.00 1.63 (1.13,2.35)	1.00 1.02 (0.66,1.58)
Family Hx of mental illness	Yes No	95 192	45 178	1.95 (1.3,2.94) 1.00	1.89 (1.16,3.09) * 1.00
Social support	Poor Moderate Strong	141 117 29	73 123 27	1.79 (0.99,3.26) 0.88 (0.49,1.58) 1.00	1.48 (0.76,2.89) 0.87 (0.45,1.65)
Anxiety	Yes No	202 85	87 136	3.71 (2.56,5.37) 1.00	2.76 (1.8,4.2) ** 1.00
Depression	Yes No	85 202	27 196	3.05 (1.89,4.91) 1.00	1.93 (1.11,3.3) * 1.00

*p-value< 0.05, **p-value< 0.01, model goodness of fit (Hosmer and Lemeshow) (*p* = 0.92).

Hx: history.

In multivariable analysis, being divorced, having depressive and anxiety symptoms, and having a history of mental illness were significantly associated with poor sleep quality among HIV-negative postpartum women at a *p*-value of<0.05. The odds of reporting poor sleep quality among divorced HIV-negative postpartum women were 7.4 times higher compared to married women [AOR =7.4, 95% CI (1.85, 29)]. Poor sleep quality among HIV-negative postpartum women who had depressive symptoms was 2.67 times higher than those without depressive symptoms [AOR =2.67, 95% CI (1.66, 4.31)]. HIV-negative women who had anxiety symptoms were more than two times at risk of reporting poor sleep quality compared to those who did not have anxiety symptoms [AOR =2.14, 95% CI (1.33, 3.44)]. Having a family history of mental illness was another factor found to be associated with poor sleep quality among HIV-negative women [AOR = 1.86, 95% CI (3.28, 9.12)].

In multivariable analysis, poor social support, anxiety, and depressive symptoms were significantly associated with poor sleep quality among HIV-positive postpartum women at a *p*-value of<0.05.

Among HIV-positive postpartum women, the likelihood of experiencing poor sleep quality was more than five times higher in those who had anxiety symptoms than their counterparts [AOR = 5.2 with 95% CI (1.40, 19.5)]. The likelihood of having poor sleep quality was six times increased among women who had poor social support compared to those with strong or moderate social support [AOR = 6, 95% CI (1.63, 22.4)]. The likelihood of reporting poor sleep quality among women who had depressive symptoms was higher compared to women who did not have depression [AOR = 4.9, 95% CI (1.3, 18.29)] (**Table 7**).

**Table 7:** Bivariable and multivariable analysis of factors associated with poor sleep quality among HIV-positive and negative women attending PNC follow-up at Comprehensive Specialized Hospitals in Addis Ababa, 2023 (*n* = 510).

Variables and categories	HIV-negative women (*n* = 410)				HIV-positive women (*n* = 100)			
	Poor sleep quality		95% CI		Poor sleep quality		95% CI	
	Yes	No	COR	AOR	Yes	No	COR	AOR
Marital status								
Single	24	21	1.25 (0.67, 2.34)	0.67 (0.33,1.38)				
Married	157	172	1.00	1.00				
Widowed	9	7	1.4 (0.51, 3.87)	0.69 (0.22, 2.22)				
Divorced	17	3	6.2 (0.78, 21.58)	7.4 (1.85,29)*				
Education								
No education					18	2	7.7 (1.24,47.7)	2.3 (0.27, 19.5)
Primary school					28	7	3.4 (0.87,13.5)	2.5 (0.46, 12.9)
Secondary school					27	5	4.6 (1.08,19.7)	5.2 (0.82, 33)
Diploma and above					7	6	1.00	1.00
Occupation								
Student	14	12	1.48 (0.62, 3.54)	2.12 (0.76, 5.92)				
Self-employed	43	36	1.5 (0.83, 2.76)	1.38 (0.68, 2.8)				
Government employed	48	42	1.44 (0.81, 2.59)	1.14 (0.57, 2.24)				
Unemployed	61	61	1.27 (0.74, 2.18)	1.32 (0.71, 2.46)				
Housewife	41	52	1.00	1.00				
Social support								
Poor					55	6	0.19 (0.67,0.57)	6 (1.63, 22.4)*
Moderate/strong					25	14	1.00	1.00
Depression								
Yes	95	148	3.17 (2.1,4.79)	2.67 (1.33,3.44)**	52	5	5.6 (1.83,16.93)	4.9 (1.3, 18.29)*
No	112	55	1.00	1.00	28	15	1.00	1.00
Anxiety								
Yes	135	78	3 (2, 4.49)	2.14 (1.33,3.44)**	68	9	6.9 (2.36,20.26)	5.2 (1.40, 19.5)*
No	72	125	1.00	1.00	11	12	1.00	1.00
Having CMI								
Yes	58	45	1.37 (0.87, 2.14)	1 (0.62, 1.76)				
No	149	158	1.00	1.00				
FHX of mental illness								
Yes	121	42	1.68 (1.25, 2.12)	1.86 (3.28, 9.12)**				
No	86	161	1.00	1.00				
Pregnancy								
Wanted	118	138	1.6 (1.07,2.39)	0.75 (0.45, 1.25)				
Unwanted	89	65	1.00	1.00				

*p-value< 0.05, **p-value< 0.01, model goodness of fit (Hosmer and Lemeshow) (*p* = 0.84). FHx, family history; CMI, chronic medical illness other than HIV/AIDS. Bold indicates total risk factors associated with the outcome variable.

## Discussion

Poor sleep quality is a frequent postnatal mental health issue affecting both the mothers’ and the children’s lives that needs to be addressed as soon as possible. The purpose of this study was to determine the prevalence and factors that contribute to poor sleep quality. Additionally, it evaluated the magnitude of poor sleep quality among HIV-positive and -negative postpartum women.

In this study, poor sleep quality prevalence among HIV-negative and -positive postpartum mothers was 50.5% and 80%, respectively. This discrepancy could be the result of the virus’ effects, which have been linked to poor sleep efficiency, early morning awakenings, numerous nighttime awakenings, and increased sleep onset latency among HIV-positive individuals (39, 40). People with the virus experience sleep difficulties due to physical symptoms such as pain, stomach cramping, diarrhea, incontinence, itching, burning, fever, night sweats, coughing, and dyspnea (39). Furthermore, the HIV’s effect on CNS, opportunistic infections due to compromised immunity, medication side effects like efavirenz (EFV), and vulnerabilities to mental health problems in people living with the virus can adversely affect their sleep quality compared to HIV-negative women (20, 26, 27).

This study revealed that the overall prevalence of poor sleep quality among postpartum (HIV-positive and negative) women at Comprehensive Specialized Hospitals in Addis Ababa was 56.27% [95% CI (51.9, 60.6)]. The findings of this study were in line with studies in Norway (57.7%) (41), northern Taiwan (60%) (42), and South America (59%) (43). However, the results of this study were higher than studies in Ethiopia (21.8% (17), 24% (18), and 39.4% (19)) and South Korea (48.4%) (44). They are also higher than studies in Hans, China (29.57%) (45) and Vietnam (41.2%) (46). The possible reason for this discrepancy could be due to differences in the measurement tool used and the cutoff point of PSQI, sample size difference, and variations in study participants, e.g., studies in Vietnam and Ethiopia were conducted among pregnant women. Healthcare delivery system variations might have brought this difference. Furthermore, this finding may also be higher since the current study included women with HIV, who have reduced quality of sleep due to the viral infection (47).

The prevalence of poor sleep quality in the current study was lower than that of studies conducted in China (67.2%) (11), Iran (62.5%) (48), Germany (83.6%) (49), and Taiwan (87.5%) (6). This variation could be due to tool differences and sociocultural variations wherein Ethiopian social interactions are widely practiced, and special attention is given to postpartum women (50). These active social rituals might have helped women to have better sleep quality in this study compared to countries where an individualized life is widely practiced. Besides this, there is a cultural practice in China called “doing the month” in which new mothers’ time is spent recuperating in bed, avoiding domestic tasks and habits like watching TV and reading books, as these activities are thought to be linked to illnesses in later life. House confinement with the windows closed and avoiding contact with the outside world for 1 month decreases social interaction (51). One of the determinants of poor sleep quality among postpartum women was HIV status. The odds of reporting poor sleep quality among HIV-positive women were higher compared to those of HIV-negative women. This could be due to the entry of the virus into the CNS, which activates macrophages and astrocytes, thus decreasing the release of chemicals that regulate sleep (52, 53). Additionally, raising a child can be stressful, and worrying about the child’s health in relation to the virus makes women’s lives more difficult (54). Being divorced was also associated with the outcome variable among both HIV-negative and -positive postpartum women. This finding was supported by a study conducted in Ethiopia (18). This might be due to the fact that being a single mother could lead to distress and increase the burden of responsibilities, which indirectly affects the subjective sleep quality of women (55). Being divorced is also a risk factor for developing depression, which has a negative impact on sleep quality (2).

This study showed that poor social support was associated with poor sleep quality among HIV-positive women. This result supported a study carried out in Ethiopia (17). Instrumental, emotional, and psychological support is a crucial factor that women need during the postpartum period and lacking this could lead them to despair and distress (56). Furthermore, the lack of husband’s support during the postpartum period, such as not giving them enough attention, letting them handle the baby’s care and working alone, and being unable to encourage and support them, could lead mothers to worry about the burden of responsibilities, thus affecting their sleep status (57).

Having a family history of mental illness was also a contributing factor to poor sleep quality in this study, and this evidence was supported by different studies (17, 18). A family history of mental illnesses is one risk factor for mental disorders (the hereditary effect), notably mood disorders, which carry a risk of between 10% and 25% for a child if one parent has a mood disorder (2). Moreover, taking care of a family member who is mentally ill may provide additional stress, which raises the occurrence of mental distress that may lead to sleep problems (58).

The odds of developing poor sleep quality was higher in participants with depression compared to their counterparts. This evidence was revealed by other studies (17, 59, 60). Mothers with depressive symptoms experience mood instability, lack of confidence, a sense of misfortune, and low self-esteem, all of which increase the likelihood of having trouble falling asleep (8). Anxiety was also another predictor variable among postpartum women affecting their sleep quality. This finding was also revealed by studies done in Ethiopia (26, 61). This could be due to anxious people experiencing slower sleep start, more awakenings, and longer stretches of awake time at night, with fewer transitions into non-REM sleep (62).

### Strengths and limitations of the study

The current study assessed the prevalence of poor sleep quality and its determinants among postpartum women, comparing HIV-positive and -negative groups, and employed validated assessment tools that reveal its strengths. However, using an interviewer-administered questionnaire may be affected by social desirability and recall bias, and the nature of the study design may not establish cause–effect relationships between the dependent and independent variables.

### Implications of the study

The postpartum period is a critical time in a woman’s life that comes with different stresses and responsibilities. Therefore, assessing the burden of poor sleep quality and its determinants among postpartum women can be helpful for healthcare providers working in the area to improve maternal mental health by addressing the identified factors. Both governmental and non-governmental organizations can implement the findings of this study in developing strategies to tackle the problem. The findings of this study may also serve as baseline information for future researchers to conduct longitudinal studies to further investigate the temporal relationship of the covariate and outcome variable.

## Conclusions and recommendation

The prevalence of poor sleep quality among HIV-positive postpartum women was higher compared to their HIV-negative counterparts. Having comorbid anxiety symptoms and poor social support were factors associated with poor sleep quality among HIV-positive postpartum women, whereas being divorced, having a family history of mental illness, poor social support, comorbid anxiety, and depression symptoms were also factors contributing to poor sleep quality among HIV-negative women.

Therefore, it is advised that health professionals working at postnatal care clinics should routinely screen postpartum women for sleep disturbances and pay particular attention to women who are divorced, have a family history of mental illness, receive poor social support, and experience symptoms of depression and anxiety. Additionally, collaboration between postnatal care providers and mental health professionals would be beneficial for the early detection and prompt intervention of sleep-related and other mental health issues. It is also recommended for future researchers to conduct longitudinal studies to investigate the cause–effect relationship of the variables.

## Data Availability

The datasets presented in this article are not readily available because they contain sensitive information. Requests to access the datasets should be directed to the corresponding author.
